# Effects of Aqueous Extract of Three Cultivars of Banana *(Musa acuminata)* Fruit Peel on Kidney and Liver Function Indices in Wistar Rats

**DOI:** 10.3390/medicines4040077

**Published:** 2017-10-23

**Authors:** Chidi Edenta, Stanley I. R. Okoduwa, Oche Okpe

**Affiliations:** 1 Department of Biochemistry, Renaissance University, Ugbawka, Enugu State 402212, Nigeria; chidiedenta@gmail.com; 2 Directorate of Research and Development, Nigerian Institute of Leather and Science Technology, Zaria, Kaduna State 810221, Nigeria; 3 Infohealth Awareness Unit, SIRONigeria Global Limited, Abuja, Federal Capital Territory 900288, Nigeria; 4 Department of Biochemistry, Federal University of Agriculture, Makurdi, Benue State 970101, Nigeria; ocheking10@gmail.com

**Keywords:** banana, *Musa sapientum*, hepatotoxicity, nephrotoxicity, wistar rat, rats

## Abstract

**Background:** *Musa acuminata* fruit peels are used in the northern part of Nigeria for the treatment of hypertension and other cardiovascular related diseases. The effects of aqueous extracts of ripped fruit peel of three cultivars of *Musa acuminata* (*Saro, Ominni* and *Oranta*) on the hepatic and renal parameters of normal rats were examined. **Methods:** Fruit peel aqueous extracts (FPAE) of the 3 cultivars of Bananas (100 mg/kg b.w.) were administered by oral intubation (that is through esophageal cannula) to normal rats (140–180 g) for a period of 28 days. Blood samples were collected for determination of plasma aspartate amino transferase (AST), alanine amino transferase (ALT), alkaline phosphatase ALK-P), total protein, albumin, creatinine as well as urea. **Results:** From the results obtained, there were no significant (*p* < 0.05) changes in the ALK-P, AST, ALT, total protein and albumin among the experimental rats administered FPAE of the 3 cultivars of *Musa acuminata* when compared with the normal control group. There was a significant (*p* < 0.05) increase in the level of serum creatinine (in mg/dL) (1.53 ± 0.23) when compared to the normal control (0.72 ± 0.15), *Ominni* (0.92 ± 0.39) and *Oranta* (0.74 ± 0.22). Similarly, there was a significant (*p* < 0.05) increase in the level of serum urea (in mg/dL) of *Saro* (41.56 ± 4.68) when compared to the normal control (26.05 ± 0.73), *Ommini* (28.44 ± 2.43) and *Oranta* (26.10 ± 2.94). **Conclusion:** The findings reveal the *Saro* cultivar of *Musa acuminata* to be nephrotoxic and not a good potential drug candidate among the cultivars studied hence should be discouraged in the treatment of hypertension and other cardiovascular related diseases.

## 1. Introduction

The liver plays a central role in the metabolism of drug, xenobiotics, protein synthesis and in maintaining biological equilibrium in organisms [1]. Although the liver is the target for most xenobiotics, the kidney usually shares in the burden of xenobiotic exposure, and the two organs carry out most of the biotransformation of xenobiotics [2]. Herbal drugs play a role in the management of various liver disorders, most of which speed up the natural healing processes of the liver [3]. Since ancient times, people have been exploring nature, particularly plants, in search of new drugs, and this has resulted in the use of a large number of medicinal plants with curative properties to treat various diseases [4]. The popularity and availability of the traditional remedies have generated concerns regarding the safety, efficacy and the responsibility of practitioners using traditional remedies [1]. Banana is one such plant that has gained popularity in treatment of various ailment.

Banana, generally known as *Musa sapientum*, is a familiar tropical fruit in the world. It originated mainly from intra- and inter-specific hybridizations between two wild diploid species, *M. acuminata* Colla (‘A’ genome) and *M. Balbisiana* Colla (‘B’ genome) [5]. Banana is a treelike perennial herb that grows 5–9 m in height, with a tuberous rhizome and a hard, long pseudo-stem. The inflorescence is big, with a reddish-brown bract, and is eaten as a vegetable. The ripe fruits are sweet, juicy and full of seeds, and the peel is thick [6,7]. Banana is the most popular fruit in industrialized countries. It is cultivated in over 130 countries as the second-most produced fruit after citrus [8,9]. It contributes approximately 17% of the world’s total fruit production [8]. It is one of a good number of consumed fruits in tropical and subtropical regions of the world [10]. The scientific names of most cultivated bananas are *Musa acuminata*, *Musa balbisiana*, and *Musa* × *paradisiaca* for the hybrid *Musa acuminata* × *M. balbisiana*, depending on their genomic constitution. The old scientific name *Musa sapientum* is no longer used. The classification of cultivated bananas has long been a problematic issue for taxonomists. Bananas were originally placed into two species based only on their uses as food: *Musa sapientum* for dessert bananas and *Musa paradisiaca* for plantains. Series of papers published from 1947 onwards showed that *Musa sapientum* and *Musa paradisiaca* were actually cultivars, and were descendants of two wild seed-producing species, *Musa acuminata* and *Musa balbisiana*. In Nigeria, some of the most common edible banana cultivars include *Musa paradisiaca*, *Musa acuminata* (Cavendish banana) (MAC), and *Musa acuminata* (Red Dacca) (MAR) [11]. The cultivated varieties can present different genomic combinations: AA, AB, AAA, AAB, ABB, AAAA, AAAB, AABB and ABBB, diploids, triploids and tetraploids [5,12]. South-western Nigeria often blends the dried *Musa* spp. peels with the yam flour, as part of their stable foods [7].

The peels of bananas, constitute up to 35% of the ripe fruit, and are regarded as household and industrial food waste, being discarded in large quantities [13]. Taking a piece of banana peel and placing it on a wart, with the yellow side out, can be a natural alternative for killing off a wart; rubbing the affected area of a mosquito bite with the inside of a banana skin reduces swelling and irritation [14]. The peels of ripe bananas can be used to make a poultice for wounds, which is wrapped around an injury to reduce pain or swelling [15]. Banana tree has an important local and traditional value for treating anemic people. They are regarded as healthy food for children from around six months of age, because it does not produce cramps or diarrhea [16]. The ripe fruits/or pseudo-stems of bananas are used to treat diarrhea. The juice from *Musa* spp., is used to treat abdominal pain [16,17]. Unripe banana peels are used as a food source in scientific research, owing to their chemical composition, which was first described by [18]. Bioactive compounds such as alkaloids, anthocyanins, flavonoids, glycosides, phlobatannins, tannins, and terpenoids have been reported in banana peels, and these compounds have been shown to exert various biological and pharmacological effects (antibacterial, antihypertensive, antidiabetic, and anti-inflammatory activities) [16]. For the purpose of this research work, only three of the genomic combinations of *Musa acuminata* were used, which are the ones distributed within Nigeria. The cultivars are AA, AAB and ABB. Locally they are called “*Saro*”, “*Paranta/Oranta*” and “*Amina/Ominni*” respectively [12].

*Musa acuminata* fruit peels are used in the northern part of Nigeria for the treatment of hypertension and other cardiovascular related diseases [5]. Despite that several scientific studies that have validated the folkloric uses of different parts of the *banana,* there is no study in the open scientific literature that has provided scientific evidence for the claimed use of the peel of *Musa acuminata* in the management of cardiovascular related diseases. Additionally, no research has been done to determine the effects of its fruit peels on hepatic and renal parameters in rats. Therefore, this study was aimed at evaluating the effects of aqueous extract of three cultivars of *Musa acuminata* fruit peel on kidney and liver function indices in wistar rats.

## 2. Materials and Methods 

### 2.1. Plant Sample Collection and Identification 

The ripe banana fruit peels of the species (cultivars) *Saro, Oranta* and *Ominni* were collected from their natural habitat within Samaru (located on latitude: 11°9′55.3′′ N and longitude: 7°39′5.84′′ E), Zaria area of Kaduna State, Nigeria [19]. They were identified at the herbarium unit of the Biological Sciences Department, Ahmadu Bello University, Zaria Nigeria.

### 2.2. Experimental Protocols

Twenty wistar albino rats of both sexes weighing 140–180 g were purchased from the Faculty of Pharmaceutical Sciences, Ahmadu Bello University, Zaria-Nigeria. They were housed in polypropylene cages in a room where a congenital temperature was 27 °C ± 1 °C and 12 h light and dark cycles were maintained. The animals were allowed to acclimatize to the environment for fourteen days. During this period they were supplied with a commercial growers mash (Grand cereals limited, Jos, Nigeria) available in pellet form and water *ad libitum*.

### 2.3. Ethical Consideration

The study was approved on 18th September, 2014 by the Institutional animal care and use committee (Protocol number; AREC/EA14/273). All protocols were in accordance with the ethical committee guidelines that are in compliance with the National and International Laws and Guidelines for Care and Use of Laboratory Animals in Biomedical Research [20,21].

### 2.4. Period of Experimentation

The research was conducted between November 2014 and August 2015.

### 2.5. Preparation and Extractions of Plant Parts

The banana peels were air-dried in the laboratory for a period of two weeks and then made into powder by grinding and sieved with a mesh size of 0.05 mm. The banana peels aqueous extracts were prepared by soaking 300 g of the powder in 1500 mL (1:5) distilled water in a 2 L conical flask [22]. It was stirred and allowed to stand for 48 h. The extracts were thereafter filtered using filter paper. The filtrates were concentrated to dryness on a water bath set at 45 °C.

### 2.6. Acute Toxicity (LD_50_) Test

The mean lethal dose of aqueous peel extracts of *Oranta, Ominni* and *Saro* of *M. acuminata* was determined in albino rats using the method described by Lorke [23]. The LD_50_ was conducted in a pilot study using 9 Wistar rats. The rats were randomly divided into 3 groups of 3 rats each and 10, 100, and 1000 mg/kg b.w., respectively, was administered orally. The animals were monitored for behavioral changes and mortality for 24 h. When no death was observed in any of the groups, 5 other groups were given 1250, 1500, 2000, 2500 and 5000 mg/kg b.w. of the extract and monitored for 24 h for changes in behavior and mortality. The LD_50_ is generally calculated as the geographical mean of the least lethal dose that killed a rat and the highest dose that did not kill a rat. Usually, the extract dose administered to the animal is either calculated as 1/10th of the observed acute toxicity test or extrapolated from the human dose. In this study, the reference body surface area of rat (0.02 m^2^) [24] was multiplied by the maximum dose (5000 mg/kg b.w.) tested during the acute toxicity study [25]; that is, 0.02 × 5000 = 100.

### 2.7. Animal Grouping and Treatment

A total of 20 rats were used. The rats were divided into 4 groups of 5 rats each as follows:

Group I: Normal control received feed and distilled water only for 28 days.
Group II: Normal rats treated with *Saro* 100 mg/kg bw/day aqueous extract orally for 28 days.
Group III: Normal rats treated with *Ominni* 100 mg/kg bw/day aqueous extract orally for 28 days.
Group IV: Normal rats treated with *Oranta* 100 mg/kg bw/day aqueous extract orally for 28 days.

### 2.8. Blood Sample Collection

At the end of the experimental period, the rats were sacrificed by anesthesia using chloroform before sample collection through cardiac puncture. Blood was collected into EDTA bottles and centrifuged at 3500 rpm for 10 min and the clear sera aspirated off for biochemical evaluation.

### 2.9. Biochemical Analysis

Alanine aminotransferase (ALT) and aspartate aminotransferase (AST) were determined by monitoring the concentration of pyruvate hydrazone formed with 2,4, dinitrophenyl hydrazine and oxaloacetate hydrazone formed with 2,4-dinitrophenyl hydrazine respectively using Randox Diagnostic kits (Randox laboratories Ltd., Antrim, UK) [26]. Serum Creatinine and urea were assayed using standard procedures as described by Varley and Alan [27].

### 2.10. Statistical Analysis 

Data obtained were expressed as mean ± SD. The data were analyzed using analysis of variance (ANOVA). The difference between the various extracts and animal groups were compared using the Duncan Multiple Range Test. The values of *p* < 0.05 were considered as statistically significant.

## 3. Results and Discussion

In the acute toxicity test study, no death was recorded even at a dose of 5000 mg/kg b.w. This signifies that the LD_50_ is greater than 5000 mg/kg b.w., hence there was nothing to present as results under this section. This observation was in agreement with the reports of Ezekwesili et al. [28]. According to the Hodge and Sterner [29] toxicity scale, *Musa acuminata* is said to be in the non-toxic herbal drug category [30]. The changes in serum liver marker enzymes and proteins of Wistar rats administered with extracts of *Musa acuminata* are shown in Table 1 and Table 2, respectively. The results from this study show that there were no significant (*p* > 0.05) changes in the levels of aspartate amino transferase, alanine aminotransferase, alkaline phosphatase, total protein and albumin of all the extract-treated groups when compared to the normal control group. As shown in Table 3, there was a significant (*p* < 0.05) increase in the creatinine level of the group treated with the extract of *Saro* (1.53 ± 0.23) when compared with *Oranta* (0.74 ± 0.22), *Ominni* (0.92 ± 0.39) and normal control (0.72 ± 0.15) groups. Similarly, there was a significant (*p* < 0.05) increase in the level of serum urea of *Saro* (41.56 ± 4.68) when compared to the normal control (26.05 ± 0.73), *Ominni* (28.44 ± 2.43) and *Oranta* (26.10 ± 2.94) groups.

The liver is the major organ of xenobiotic metabolism and detoxification, which makes it vulnerable to hepatotoxicity [31]. AST and ALT are key enzymes involved in the breakdown of amino acids into α-keto acid, which are routed for complete metabolism through the Krebs cycle and electron transport chain. They are regarded as precise biomarkers for liver damage. Also, changes in membrane-bound alkaline phosphatase (ALP) affects membrane permeability and produces derangement in the transport of metabolites [32]. Any damage on the liver results in the release of these marker enzymes into the system above a certain threshold [3]. From the results of our study as indicated in Table 1, it was observed that there were no significant changes (*p* < 0.05) in the levels of serum ALT, AST and ALP of all the extract treated groups when compared to normal control group. These observed insignificant (*p* > 0.05) changes are in accordance with good functioning of the liver. This implies that the peel extracts are not hepatotoxic. Concentrations of albumin and total protein in the serum can be used to assess the health status of the liver and can also be used to ascertain different type of liver damage [33,34,35]. The liver is the sole site for the synthesis of albumin, which makes up approximately 60% of serum protein concentration [36]. The present study revealed (Table 2) that there were no significant (*p* > 0.05) changes in total protein and albumin levels of the extract treated groups as compared to the normal control rats. The data obtained with respect to liver function indices indicate no cellular toxicity of the extracts on the liver of the experimental rats. However, as presented in Table 3, there was an increase in serum creatinine concentration (1.53 ± 0.23 mg/dL), reflecting a failed capacity of the kidney to effectively excrete creatinine. By inference, it implies that the ability of the two kidneys to effectively excrete creatinine, might have been exceeded. Serum creatinine and urea are common biomarkers for prediction of renal dysfunction, due to the fact that they are elevated considerably when there is a dysfunction of the kidney [33]. The level of serum creatinine usually doesn’t rise until at least half of the nephrons of kidney are destroyed or damaged [37]. For differential diagnosis, simultaneous determination of serum creatinine along with urea is a standard practice; hence, serum urea was also estimated. Though urea is inferior to other markers, such as creatinine, blood urea is grossly influenced by other factors, such as diet and nutrition [38]. The marked significant increase in urea (41.56 ± 4.68) of *Saro* means that its intake may pose an undesirable effect on the kidney. Since the elevated levels of urea and creatinine are markers of kidney function [38,39], it then indicates that the extract of *Saro* peel cultivar may impair renal function at the comparative human dose levels.

## 4. Conclusions and Recommendation

The results from this study revealed that rats treated with the aqueous extracts of the three cultivars of *Musa acuminata* pose no threat to the liver. Conversely, aqueous extracts of *Saro* cultivars showed increase in the biomarkers of the kidney. Thus, intake of peel extracts of *M. acuminata,* particularly the *Saro* cultivar, as a drug might lead to potential kidney problems in the management and/or treatment of hypertension and other cardiovascular related diseases. At present, the exact mechanism of action of *M. acuminata* is not fully known. On this note, further investigations in this direction are needed for possible isolation and structural elucidation of the components of *M. acuminata.*

## Figures and Tables

**Table 1:** Effect of *Musa acuminata* peels on some Serum Liver Marker Enzymes concentration in rats.

Groups (*n* = 5)	Serum ALK-P (U/L)	Serum ALT (U/L)	Serum AST (U/L)
NC	64.40 ± 15.93 ^a^	43.53 ± 6.11 ^a^	21.65 ± 5.77 ^a^
N + OMN_100_	36.80 ± 14.74 ^a^	52.67 ± 8.32 ^a^	25.01 ± 8.66 ^a^
N + ORT_100_	61.07 ± 10.16 ^a^	41.33 ± 2.31 ^a^	23.34 ± 7.64 ^a^
N + SRO_100_	46.03 ± 16.00 ^a^	45.46 ± 9.24 ^a^	26.67 ± 2.89 ^a^

Values are means of five determination ± SD; ^a^ Values with different superscripts down the column are significantly different (*p* < 0.05); NC: Normal rats Control, N + SRO_100_: Normal rats + Saro Extract (100 mg/kg), N + ORT_100_: Normal rats + Oranta Extract (100 mg/kg), N + OMN_100_: Normal rats + Ominni Extract (100 mg/kg), ALK-P: Alkaline Phosphatase, AST: Aspartate aminotransferase, ALT: Alanine aminotransferase.

**Table 2:** Effect of aqueous extracts of *Musa acuminata* peels on serum total protein and albumin concentration in rats.

Groups (*n* = 5)	Total Protein (g/L)	Albumin (g/L)
NC	56.95 ± 6.84 ^a^	31.16 ± 6.31 ^a^
N + OMN_100_	64.46 ± 10.75 ^a^	27.57 ± 2.07 ^a^
N + ORT_100_	65.23 ± 9.54 ^a^	26.77 ± 3.30 ^a^
N + SRO_100_	60.25 ± 7.18 ^a^	23.97 ± 3.74 ^a^

Values are means of five determination ± SD. ^a^ Values with different superscripts down the column are significantly different (*p* < 0.05) NC: Normal rats Control, N + SRO_100_: Normal rats + *Saro* Extract (100 mg/kg), N + ORT_100_: Normal rats + *Oranta* Extract (100 mg/kg), N + OMN_100_: Normal rats + *Ominni* Extract (100 mg/kg).

**Table 3:** Effect of aqueous extracts of *Musa acuminata* peels on serum creatinine and urea concentration in rats.

Groups (*n* = 5)	Creatinine (mg/dL)	Urea (mg/dL)
NC	0.72 ± 0.15 ^a^	26.05 ± 0.73 ^a^
N + OMN_100_	0.92 ± 0.39 ^a^	28.44 ± 2.43 ^a^
N + ORT_100_	0.74 ± 0.22 ^a^	26.10 ± 2.94 ^a^
N + SRO_100_	1.53 ± 0.23 ^b^	41.56 ± 4.68 ^b^

Values are means ± SD of *n* = 5 determinations. ^a^ Values with different superscripts down the column are significantly different (*p* < 0.05), NC: Normal rats Control, N + SRO_100_: Normal rats + *Saro* Extract (100 mg/kg), N + ORT_100_: Normal rats + *Oranta* Extract (100 mg/kg), N + OMN_100_: Normal rats + *Ominni* Extract (100 mg/kg).
